# Interaction of the Antimicrobial Peptide Polymyxin B1 with Both Membranes of *E*. *coli*: A Molecular Dynamics Study

**DOI:** 10.1371/journal.pcbi.1004180

**Published:** 2015-04-17

**Authors:** Nils A. Berglund, Thomas J. Piggot, Damien Jefferies, Richard B. Sessions, Peter J. Bond, Syma Khalid

**Affiliations:** 1 School of Chemistry, University of Southampton, Highfield, Southampton, United Kingdom; 2 Bioinformatics Institute (A*STAR), Singapore; 3 School of Biochemistry, Bristol, United Kingdom; 4 Department of Biological Sciences, National University of Singapore, Singapore; University of Uppsala, SWEDEN

## Abstract

Antimicrobial peptides are small, cationic proteins that can induce lysis of bacterial cells through interaction with their membranes. Different mechanisms for cell lysis have been proposed, but these models tend to neglect the role of the chemical composition of the membrane, which differs between bacterial species and can be heterogeneous even within a single cell. Moreover, the cell envelope of Gram-negative bacteria such as *E. coli* contains two membranes with differing compositions. To this end, we report the first molecular dynamics simulation study of the interaction of the antimicrobial peptide, polymyxin B1 with complex models of both the inner and outer membranes of *E. coli*. The results of >16 microseconds of simulation predict that polymyxin B1 is likely to interact with the membranes via distinct mechanisms. The lipopeptides aggregate in the lipopolysaccharide headgroup region of the outer membrane with limited tendency for insertion within the lipid A tails. In contrast, the lipopeptides readily insert into the inner membrane core, and the concomitant increased hydration may be responsible for bilayer destabilization and antimicrobial function. Given the urgent need to develop novel, potent antibiotics, the results presented here reveal key mechanistic details that may be exploited for future rational drug development.

## Author Summary

Antimicrobial peptides have the ability to kill harmful bacteria through interaction with bacterial membranes. This manuscript describes the first reported computational study of antimicrobial peptide interaction with both membranes of a Gram-negative bacterium. While antimicrobial peptides have been the topic of many simulation studies, these studies have not incorporated the biochemical heterogeneity of natural membranes. Our simulations add the missing biochemical details and in doing so, reveal that the mechanisms of interaction of polymyxin B1 with the inner and outer membranes of *E*. *coli*, are really rather different. The peptides insert readily into the inner membrane, whereas the interaction with the LPS-containing outer membrane is more complex. In summary our results represent a key finding for future drug development that targets these bacteria.

## Introduction

Antimicrobial peptides (AMPs) are small cationic membrane-active peptides; they can be found in most living organisms and play an essential part in innate immunity [1–3]. These peptides exhibit broad-spectrum antimicrobial activity against bacteria, fungi and viruses, making them of great biomedical interest, particularly in the field of novel antibiotic design.

Polymyxin B1 (PMB1) is a small antimicrobial lipopeptide first derived from the bacterial species *Bacilus Polymyxa* in 1947[2, 4, 5]. It is composed of a cyclic polypeptide ring and a branched fatty acid tail, and among the amino acids forming the peptide segment are the irregular amino acids D-Phenylalanine (DPhe) and α, γ-Diamino Butyric acid (DAB). Its full sequence is thus: DABC-Thr-Leu-DPhe-DAB-DABC-DAB-Thr-DAB-CO(CH_2_)_4_CH(CH_3_)CH_2_CH_3_, where DABC represents the cyclic linkage. The five non-cyclized DAB amino acids each carry a charge of +1, and thus the cationic peptide has a total charge of +5 [6]. PMB1 is a highly potent antimicrobial peptide and is selective predominantly towards all Gram-negative bacterial species, with the exception of the Proteus groups [7]. Unfortunately, treatment of patients with PMB1 has been shown to have adverse side effects on the renal and nervous system [8–10], and therefore clinical use of PMB1 has been limited to topical treatment as well as “last resort” therapy of patients infected with multidrug-resistant bacteria or with chronic conditions who suffer from recurring respiratory infections [11]. However, given the alarming rise in the number of bacterial strains exhibiting multidrug-resistance, there has recently been renewed interest in PMB1 [2].

PMB1 lipopolypeptides are known to permeate across the bacterial outer membrane (OM) by self-promoted uptake, while it is disruption of the inner membrane (IM) that subsequently leads to cell death. The peptides are thought to fulfil the initial stages of their bactericidal activity by anchoring themselves to the bacterial membrane via the DAB amino acids[12, 13]. While the precise mode of action subsequent to peptide anchoring to the membrane is still unclear, it has been established that the polypeptide ring is responsible for causing an increased permeability of the bacterial membrane[4]. It has been proposed that the observed permeabilization is caused by membrane insertion of the polypeptide ring and fatty acid tail, resulting in bilayer disruption and an outflow of intracellular components, followed by cell death [4].

Because of experimental difficulties associated with characterizing dynamic, heterogeneous systems such as membrane-bound AMPs, molecular dynamics (MD) simulations provide a complementary approach to studying their modes of action, in unprecedented detail [14, 15]. Here, we have used a series of MD simulations (Table 1) over microsecond timescales to study, the interaction of an AMP with accurate models of both membrane components of a Gram-negative bacterial cell, there has previously been only one report of a computational study of an AMP interacting with a model OM[16]. In particular, the former membrane is represented by a realistic mixture of phospholipids representative of the bacterial IM, whilst the latter is modelled as an asymmetric bilayer containing a phospholipid mixture in the inner leaflet and rough lipopolysaccharide (LPS) in the outer leaflet. For comparison, we also study the interactions of the AMP with a symmetric lipid A membrane. Computational work on AMPs is well documented [17–19] and simulations of complex models of the OM are also available [20–23], though we report one of the first combinations of the two aspects in atomistic detail. We thus investigate the molecular-level mechanisms of PMB1 binding, insertion, and bilayer disruption for both IM and OM models of the envelope of the archetypal Gram-negative bacterial species, *E*. *coli*.

**Table 1:** Details of membrane compositions & simulation lengths.

Membrane	Membrane Lipid Composition	System	Simulation Length
Re LPS Outer Membrane Model (Sim_OM)	Outer leaflet: 16 Re LPS molecules	6 PMB1 NVT (x2) (Sim_OM6)	500 ns
	Inner Leaflet: 90% PE, 5% PG and 5% Cardiolipin		
Re LPS Outer Membrane Model (Sim_OM)	Outer leaflet: 16 Re LPS molecules	6 PMB1 NPT (x2) (Sim_OM6)	500 ns
	Inner Leaflet: 90% PE, 5% PG and 5% Cardiolipin		
Re LPS Outer Membrane Model (Sim_OM)	Outer leaflet: 16 Re LPS molecules	12 PMB1 NVT (Sim_OM12)	500 ns
	Inner Leaflet: 90% PE, 5% PG and 5% Cardiolipin		
Re LPS Outer Membrane Model (Sim_OM)	Outer leaflet: 16 Re LPS molecules	9 PMB1 NPT (x2) (Sim_OM9)	500 ns
	Inner Leaflet: 90% PE, 5% PG and 5% Cardiolipin		
Re LPS Outer Membrane Model (Sim_OM)	Outer leaflet: 16 Re LPS molecules	2 PMB1 NVT (Sim_OM2)	100 ns
	Inner Leaflet: 90% PE, 5% PG and 5% Cardiolipin		
Re LPS Outer Membrane Model (Sim_OM)	Outer leaflet: 64 Re LPS molecules	6 PMB1 NVT (Sim_OMbig)	600 ns
	Inner Leaflet: 90% PE, 5% PG and 5% Cardiolipin		
Lipid A (Sim_LipA)	16 Lipid A molecules in leaflet	8 PMB1 NPT (x2) (Sim_LipA)	3000 ns
Phospholipid Inner Membrane Model (Sim_IM)	Symmetrical mixed lipid membrane 75% PE, 20% PG and 5% Cardiolipin	6 PMB1 NVT (x2) (Sim_IM6)	350 ns
Phospholipid Inner Membrane Model (Sim_IM)	Symmetrical mixed lipid membrane 75% PE, 20% PG and 5% Cardiolipin	6 PMB1 NPT (x2) (Sim_IM6)	250 ns
Phospholipid Inner Membrane Model (Sim_IM)	Symmetrical mixed lipid membrane 75% PE, 20% PG and 5% Cardiolipin	12 PMB1 NVT-1 (Sim_IM12)	355 ns
Phospholipid Inner Membrane Model (Sim_IM)	Symmetrical mixed lipid membrane 75% PE, 20% PG and 5% Cardiolipin	12 PMB1 NVT-2 (Sim_IM12)	330 ns
Phospholipid Inner Membrane Model (Sim_IM)	Symmetrical mixed lipid membrane 75% PE, 20% PG and 5% Cardiolipin	12 PMB1 NVT-3 (Sim_IM12)	60 ns
Phospholipid Inner Membrane Model (Sim_IM)	Symmetrical mixed lipid membrane 75% PE, 20% PG and 5% Cardiolipin	7 PMB1NPT (x2) (Sim_IM7)	2000 ns

## Results

### Re LPS Outer Membrane Model

To study the initial stages of AMP interaction with the envelope of Gram-negative bacteria, we simulated PMB1 in the presence of a realistic model of the asymmetric *E*. *coli* OM, composed of Re LPS in the outer leaflet and a mixture of phospholipids (including phosphatidylethanolamine, phosphatidylglycerol, and cardiolipin) in the inner leaflet. The molecular compositions of the simulated systems are given in Table 1.

#### PMB1 binding

To determine the process of PMB1 binding to the *E*. *coli* OM, we setup two independent simulations (Sim_OM6), each initially composed of six PMB1 lipopeptides randomly placed above the Re LPS leaflet. Following the initial adsorption process of all six lipopeptides over 1 microsecond of simulation, a further six PMB1 molecules were added above the LPS leaflet, and the simulations were extended for a further 0.5 microsecond (Sim_OM12). Three of these additional PMB1 peptides became bound to the membrane during this period, resulting in a nine-PMB1-bound system, which was simulated for an additional 0.5 μicrosecond (Sim_OM9).

**Fig 1:**
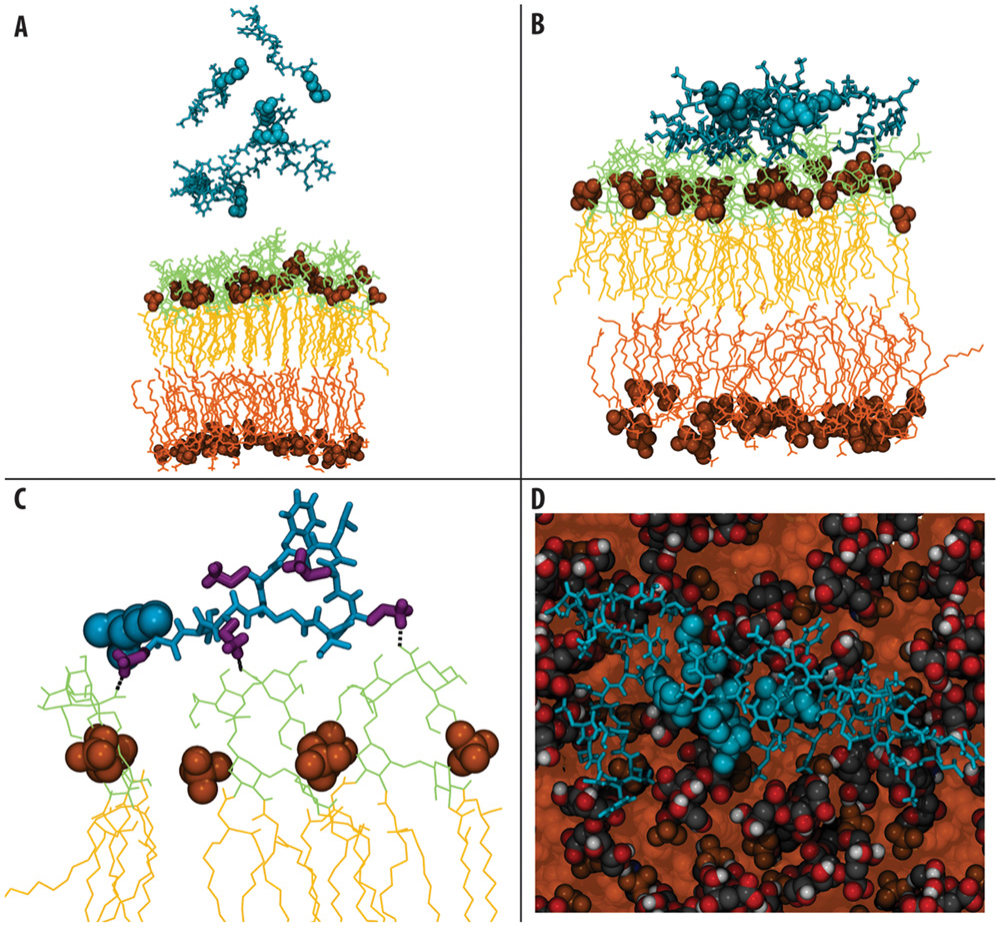
Summary of interactions with the outer membrane. A & B- Snapshots showing the starting (time = 0 microseconds) and final (time = 2 microseconds) configurations of the asymmetric membrane model systems. (PMB1 non- tail regions: cyan, licorice format, PMB1 tails: cyan, VdW format, lipid phosphate groups: orange, VdW format, LPS sugars: lime, lines format, LPS phospholipids: orange, lines format). C- Hydrogen bonding interactions between PMB1 and the LPS sugars, colored as above with DAB residues: purple, licorice format. D- Aggregation of PMB1 on the LPS surface, colored as above, but with LPS tails: orange, VdW format

In each of the simulations, the first peptides were observed to interact with the LPS sugars within only a few nanoseconds, where interaction is defined as atoms ≤0.35 nm apart, (Fig 1). As demonstrated by the sudden drop in solvent accessible surface area, and by monitoring the mean centre of mass coordinate of the peptides along the z-axis (i.e. membrane normal) (S1 Fig and S2 Fig), the majority of the PMB1 molecules became bound within ~200 ns. Moreover, the component centre of mass analysis reveals that the DAB residues are largely responsible for the initial binding process, acting as a “landing platform” prior to the remainder of the PMB1 adsorption process, consistent with previously reported fluorescence studies (S3 Fig) [12, 13].

As the only fully ionized residue in PMB1, DAB is primarily responsible for the electrostatic interactions that are key to AMP binding, interacting with the negatively charged phosphates [16, 24]. The DAB side chain alone accounts for 43% of the total number of hydrogen bonds formed (S1 Table) between any part of the peptide and the LPS sugars during the initial 300 ns of simulation, despite only making up 25% of the total number of atoms in PMB1. Moreover, during the last 500 ns of these simulations, the DAB residues were responsible for over 50% of the hydrogen bonds formed between the whole PMB1 and the LPS sugars.

#### PMB1 aggregation

We did not observe spontaneous membrane insertion of PMB1, defined as penetration of PMB1 into the acyl tails region of the bilayer, although we did observe insertion of DAB residues between the sugar head groups of LPS molecules. This was stabilized by PMB1-sugar hydrogen bonding interactions, with an average of ~8 hydrogen bonds per PMB1 molecule present at any one time during the final 500 ns (S2 Table). In addition, the environment immediately below the membrane surface is highly polar, thus preventing insertion driven by hydrophobicity. We observed PMB1 aggregation on the sugar head group region of the membrane surface (Fig 1) when all six of the initially-introduced PMB1 molecules had become bound to the membrane. Thus, by monitoring contacts between the PMB1 monomers (contact defined as atoms within 0.6 nm of each other, in order to include all apolar atoms that interact non-specifically), we found that their hydrophobic tails were prone to aggregating with one another. The probability of finding hydrophobic contacts between the fatty acid PMB1 tails was >75% at any one time, suggesting that these hydrophobic interactions drive aggregation, whereas the high charge density on the ring portion of the PMB1 unsurprisingly results in electrostatic repulsion between the peptide region of PMB1 molecules. This explains the observation that PMB1 aggregates adopt a micelle-like conformation within the first 100 ns on the membrane surface (Fig 1), such that the fatty acid tails of the PMB1 molecules faced towards the centre, with charged peptide rings splayed outwards away from one another. Radial distribution functions (S4 Fig) showed an increased degree of solvation in the sugar group area of the membrane, such that on average 20 water molecules were present in this region after 2 μicroseconds, compared to ~12 molecules prior to PMB1 binding. Interestingly, an additional simulation in which we reduced the PMB1 content of the system to just two peptides (Sim_OM2), also revealed aggregation/dimerisation of the peptides (S7 Fig).

Given the generally accepted idea that self-promoted uptake by PMB1 occurs then the peptides interact with the divalent cations that cross-link LPS molecules, which in turn weakens LPS-LPS interactions in the outer leaflet, enabling additional peptides to exploit these regions of weakened interactions to permeate through the outer leaflet, we performed additional simulations in which all of the Mg^2+^ ions were replaced with Na^+^ ions (maintaining an overall charge-neutral system) [25]. These simulations (Sim_OMbig), were performed to speed up the process of PMB1 insertion by prior removal of the divalent cations and were performed on simulations with larger bilayers than those discussed in the rest of the manuscript. These bilayers contained 64 LPS molecules in the outer leaflet, compared to 16 in all other simulations. Interestingly, we observed the acyl chain from one PMB1 peptide penetrate the LPS sugars and interact with the lipid A headgroup region after ~ 100 ns (S8 Fig). During the following ~400 ns it remained embedded within the headgroup region, but was unable to penetrate deeper. After ~ 500 ns the acyl chain was observed to move away from the lipid A headgroup region to interact with the sugars. The removal of Mg^2+^ ions also caused a distortion in the bilayer; increased separation between the leaflets was observed. In a further attempt to speed-up PMB1 penetration into the outer membrane we performed steered MD simulations employing pull force constants of 10 kJmol^-1^, 20 kJmol^-1^ and 50 kJmol^-1^ to avoid biasing the simulations with unphysical behaviour. The peptide was pulled consistent with experimental NMR data from *Mares et al* [26], which showed the mode of interaction of PMB1 with LPS when inserted into the membrane. After 100 ns of steered MD simulation we had still not observed insertion of PMB1 beyond the sugar groups.

#### Physical properties of the membrane

To further understand the effect of PMB1 binding upon the model OM, we monitored a number of physical properties. We measured the lateral diffusion of the lipids within the Re LPS leaflet (S3 Table) to characterise the dynamic properties of the membrane (using well established GROMACS methods [27, 28]). Before PMB1 binding, the average lateral diffusion was 0.12 (+/- 0.02) x 10^-8^ cm^2^ s^-1^, which is comparable to our previously reported simulations of pure LPS bilayers [27]. In contrast, in the final 500 ns of simulation in the presence of PMB1, the lateral diffusion had decreased to 0.08 (+/- 0.004) x 10^-8^ cm^2^ s^-1^, indicating that binding of PMB1 to the membrane restricts lipid movement. We measured the thickness of the bilayer and found that its initial value was 4.03 (+/- 0.01) nm. By the end of the simulation the membrane had thinned only slightly to 3.93 (+/- 0.11) nm (S4 Table). No obvious water penetration into the acyl tail portion of the membrane was seen as confirmed by our mass density profiles (S7 Fig). Thus, the PMB1 primarily exhibited a dynamic rather than structural effect upon membrane lipids, by interacting with and cross-linking the sugar groups.

### Lipid A Outer Membrane Model

We performed simulations of simplified OM model systems, containing lipid A in both leaflets (Sim_LipA). The reasons for doing this are two-fold; firstly this setup more easily enables the peptides to permeate into the lipid part of the membrane, allowing interrogation of these membrane-peptide interactions over a tractable timescale, and secondly it abrogates the problem of peptides moving across periodic boundaries to interact with the phospholipid portion of the asymmetric bilayer. While the lack of sugars in lipid A provides a simplified model, it does enable us to study the behaviour of the peptide at the lipid headgroup/tail interface in realistic detail. Two independent simulation systems, each comprising eight PMB1 placed ~0.5 nm above one leaflet of the membrane, were run for 3 microseconds. Here, we report only the behaviour that differs from the asymmetric bilayer studies described above.

#### PMB1 aggregation and insertion

**Fig 2:**
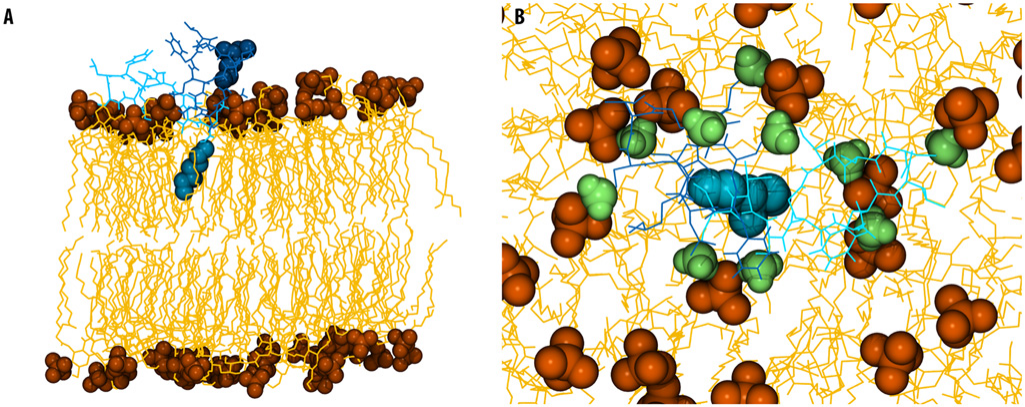
Summary of interactions with the lipid A bilayer. A- Snapshot showing one PMB1 peptide inserting into the lipid A bilayer, taken from time = 3 microseconds. (PMB1 (A) non-tail regions: cyan, licorice format, PMB1 tails: cyan, VdW format, PMB1 (B): non-tail regions: dark blue, licorice, PMB1 tails: dark blue, VdW format, lipid phosphate groups: orange, VdW format, lipid A: yellow, lines format). B- DAB residues of PMB1 parting the lipid A headgroups, colored as above with DAB in lime, VdW format.

Aggregation of PMB1 peptides was observed at the membrane surface. Interaction of the DAB residues with the membrane surface during aggregation caused water molecules to move towards the membrane. We measured an increase in solvent penetration within 1.6 nm of the membrane centre (S4 Fig), although not to the same extent as the change observed in the asymmetric OM. The major difference between this model and the asymmetric bilayer is that we observed spontaneous insertion of the fatty acid tail of one PMB1 molecule into the lipid A bilayer, ~1 microsecond after peptide aggregation. Interestingly, DAB residues of this and nearby PMB1 peptides were observed to interact with the negatively charged phosphate groups on the membrane surface, causing phosphate groups of adjacent lipids to move apart to facilitate fatty acid tail insertion into the membrane core (Fig 2).

#### Physical properties of the membrane

We observed a reduction in the lateral diffusion of the membrane lipids upon PMB1 binding, with a diffusion rate of 0.29 (+/- 0.04) x 10^-8^ cm^2^ s^-1^ in the PMB1-free state, compared to 0.065 (+/- 0.03) x 10^-8^ cm^2^ s^-1^ (S3 Table) after binding (reported diffusion rates are the combined rates calculated for both leaflets throughout this work). The sizeable error in diffusion coefficient following binding is a consequence of the variation in total number of PMB1 molecules bound to the leaflet between the two repeat simulations, but the trend in each case is the same. There was no obvious membrane thinning or water penetration into the acyl tail portion of the membrane (S7 Fig).

### Inner Membrane Model

We next turned our attention to the *E*.*coli* IM. We performed simulations of a symmetrical phospholipid membrane model (composed of phosphatidylethanolamine, phosphatidylglycerol, and cardiolipin phospolipids), and exposed it to PMB1 lipopeptides (Table 1).

#### PMB1 binding

**Fig 3:**
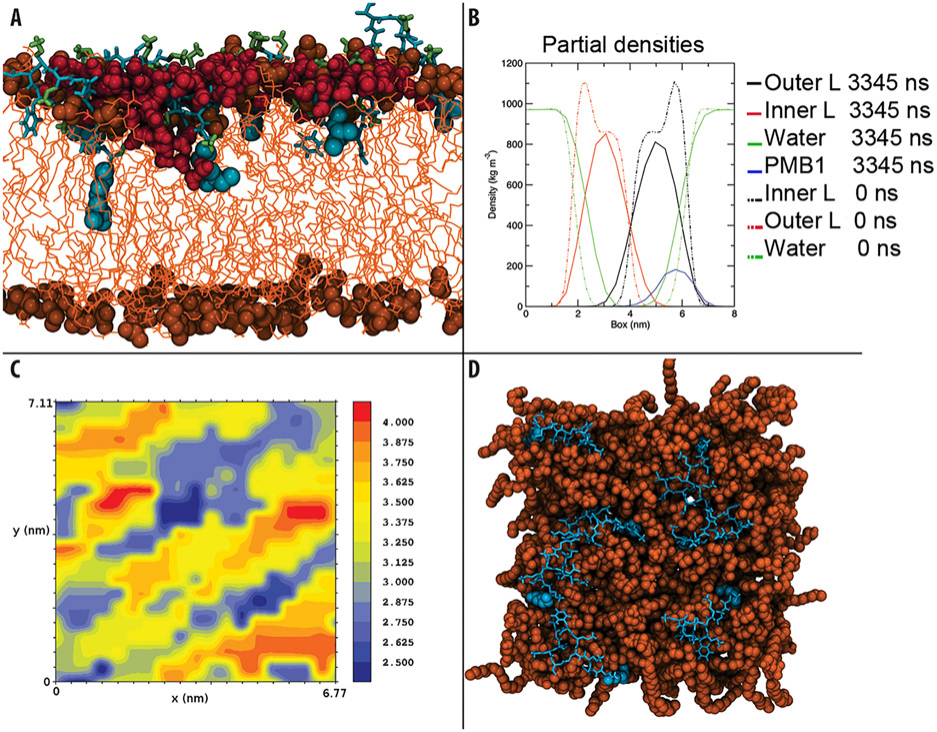
Summary of interactions with the inner membrane. A- Water penetration into the inner membrane. (PMB1 non-tail regions: cyan, licorice format, PMB1 tails: cyan, VdW format, DAB: green, licorice format, lipid phosphate groups: orange, VdW format, phospholipids: orange, lines, water: dark red, VdW format). B- Mass density of the inner membrane system, showing the difference between PMB1 free (dotted lines) system and the system after being exposed to PMB1 for 3345 ns (full lines). C & D- Bilayer thickness analysis showing the correlation between PMB1 binding regions and membrane thinning (blue represents thinner areas and red thicker areas), along with an overview of the inner membrane after 3345 ns with PMB1 present (PMB1 and membrane are colored as previously).

As with the two OM models, to monitor the process of PMB1 binding to the IM, we set up two independent simulations. Each system was comprised of six PMB1 peptides initially placed in the bulk water region at a distance of ~0.5 nm from one leaflet (Sim_OM6). In both simulations a maximum of five PMB1 molecules were observed to bind to the membrane. Remaining solvent-phase PMB1 molecules were deleted before extending the simulation to 600 ns. An additional seven peptides were then added to the system, giving an overall total of 12 peptides, (Sim_OM12), and intermittently repositioned to ease membrane binding over 3 simulations (> 745 ns). After this, a total of seven PMB1 molecules had bound, and the membrane was judged to be saturated. The unbound peptides were removed and the simulation was extended for a final 2 microseconds (Sim_IM7). Hydrogen bonding interactions were formed between the DAB residues of the peptide and the phospholipid headgroups within a few nanoseconds. As with the two OM systems, we observed that the DAB residues acted as a “landing platform” at the IM surface. The side chains accounted for 46% (S1 Table) of the total hydrogen bonds formed between the whole PMB1 molecules and the lipid headgroups. After PMB1 insertion into the membrane core however, this value dropped dramatically and DAB accounted for only 17% of hydrogen bonds formed between the entire PMB1 molecule and the lipid headgroups. This was due to spontaneous insertion of the PMB1 tail into the hydrophobic portion of the membrane, leaving a large portion of the PMB1 ring structure also buried below the lipid headgroup region, and hence unable to form hydrogen bonds (Fig 3).

#### PMB1 insertion

**Fig 4:**
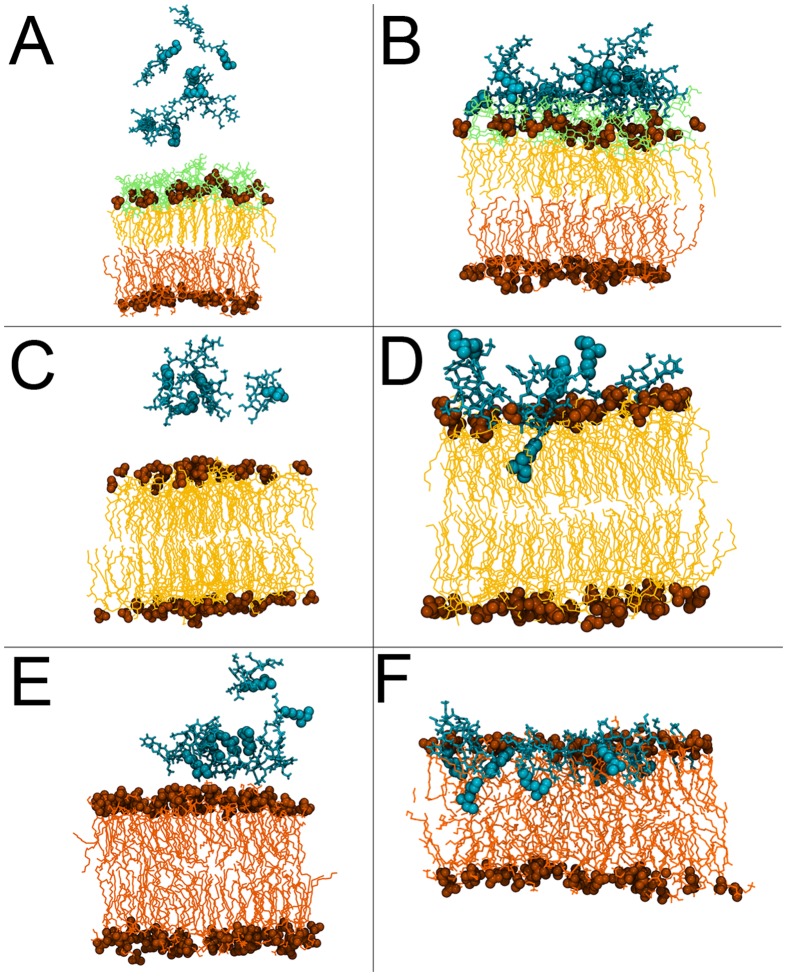
Summary of PMB1 interaction with the three membrane models. The peptides are shown at the start (A, C and E) and end of (B, D and F) simulations of the asymmetric OM, the lipid A bilayer and the symmetric IM models respectively. Insertion into the lipid A bilayer and the model inner membrane is clearly visible in the panels D and F corresponding to the end of these simulations respectively. The colour scheme is consistent with the previous figures.

Within 100 ns of binding to the membrane surface, the fatty acid tails of all of the PMB1 peptides inserted into the hydrophobic core of the bilayer. We observed that the D-Phe residues of the peptides also became embedded within the acyl tail region of the membrane within this timeframe (Fig 3). Along with the PMB1 acyl tails, these hydrophobic residues drove the insertion process, as the AMPs penetrated further into the membrane over hundreds of nanoseconds. The DAB residues were observed to drag some water molecules into the hydrophobic bilayer core (Fig 3); on average less than one water molecule was present in the PMB1 free membrane, compared to an average of seven being present during the last microsecond of simulation. Moreover, when we studied the radial distribution of solvent around DAB residues, despite being fully inserted within the non-polar acyl tail region, an average of three water molecules were still present within 0.35 nm of the DAB nitrogen atoms (Fig 3). After ~3.3 microseconds, the fatty acid tails of the PMB1 had penetrated as far down as the core of the membrane. We also noted that unlike the OM models, there was no obvious peptide aggregation in the IM model; the PMB1 peptides inserted as monomers. The interaction of PMB1 with the three membrane models is summarised in Fig 4. Quantitative analysis revealed the probability of inter-PMB1 fatty acid tail interaction was only 7% after ~3.3 μs compared to 22% during the initial 350 ns, prior to all the fatty acid tails becoming inserted.

#### Physical properties of the membrane

The lateral diffusion of lipids was clearly affected by the process of PMB1 insertion. There was an initial reduction in the rate of diffusion, from 6.62 (+/- 0.72) x 10^-8^ cm^2^ s^-1^ in the PMB1-free state, to 1.27 (+/- 0.80) x 10^-8^ cm^2^ s^-1^ or 3.56 (+/- 0.81) x 10^-8^ cm^2^ s^-1^ in the two independent simulations during the binding process, with the differences resulting from a variable number of PMB1 molecules becoming bound. However, following PMB1 tail insertion, the rate actually increased again to an average of 5.01 (+/- 0.02) x 10^-8^ cm^2^ s^-1^ (S3 Table); compared to a previously reported [27] lateral diffusion coefficient for a pure PE membrane of 3.25 (+/- 0.12) x 10^-8^ cm^2^ s^-1^. This difference between the reported values for pure PE and those calculated here is likely due to the presence of PG lipids in our model. Details of alterations in hydrogen bonding interactions within the lipids upon PMB1 insertion are provided in the SI.

We measured the impact of PMB1 on the membrane thickness. The PMB1-free membrane thickness was found to be 3.99 (+/- 0.03) nm, but at the end of our simulations the average membrane thickness was reduced to 3.28 (+/- 0.12) nm (S2 Table). As may be observed in Fig 3, regions where PMB1 became bound corresponded to those in which drastic decreases in membrane thickness were observed (S8 Fig). This is further supported by analysis of the mass density profile (Fig 3) that reveals increased water penetration, with water molecules reaching the hydrophobic core of the membrane as discussed above. We observed interdigitation of phospholipids from the inner and outer leaflets [also noticeable in Fig 3], as has been reported to occur as a result of PMB1 exposure in spin label and x-ray diffraction studies [29, 30]. Unlike in the OM systems, PMB1 binding and insertion in the IM resulted in significant increase in the disorder of the lipid acyl tails (S3 Fig).

## Discussion

Our results reveal contrasting behaviour of PMB1 in the presence of different bacterial membrane models. In the case of the LPS simulations, our observations support data from previous fluorescence studies suggesting that DAB residues are key to PMB1 binding and antimicrobial function [12, 13]. Strikingly, we observed aggregation of PMB1 on the LPS membrane surface (Fig 1 and S2 Fig), in which monomers arranged themselves in a micelle-like conformation to bury their hydrophobic tails from the polar sugar rings. This aggregation, coupled with the tendency for PMB1 to cross-link the sugar hydroxyl groups resulted in formation of an immobile, “protein membrane cluster”. The adoption of this fatty acid tail orientation and aggregation may represent a necessary prelude to pore formation, in a manner that reduces the energetic cost for translocation of the PMB1 “micelle” across the polar environment of the LPS sugar groups. Intriguingly, two attempts to speed-up the process of PMB1 insertion into the outer membrane firstly by loosening the membrane by replacing the LPS cross-linking divalent cations with monovalent ions, and secondly by performing steered MD simulations to 'pull' a peptide, also provided strong support for the hypothesis that insertion of PMB1 into the outer membrane is not a facile molecular process.

Because of the slow dynamics of the LPS system [23], we also studied a simplified OM model consisting only of lipid A. Unlike the LPS system, PMB1 appeared to have no effect upon intra-lipid A hydrogen bonding, but instead the DAB residues interacted with phosphate groups, pushing them apart, leading to local membrane deformation and creating a region of reduced membrane-surface charge density that may no longer inhibit fatty acid tail insertion. During this process, Mg^2+^ ions were intermittently displaced and rebound to phosphate groups, but no long-term ion displacement was observed. As a result, we only witnessed complete insertion of a single PMB1 molecule on the timescales feasible with atomistic simulations. Nevertheless, it has been widely documented that divalent cations are essential for maintenance of OM stability, and PMB1 has been shown to cause destabilisation via displacement of these divalent cations [6, 31, 32].

For the IM model, our results are again in agreement with previous experimental work [12, 13, 26, 33] highlighting the importance of the DAB residues in protein binding, confirming that electrostatic interactions between the DAB and the lipid headgroups are the initial driving force for PMB1 adsorption. However, in contrast with the OM, the distinguishing behaviour of the PMB1 molecules in the IM system was the lack of PMB1 aggregation as well as the striking result that the fatty acid tails of every PMB1 molecule spontaneously penetrated into the hydrophobic core of the membrane, some as far as the lower leaflet (Fig 3). Unlike the OM models, the IM exhibited a large increase in the disorder of the acyl tails upon fatty acid tail insertion. Indeed, PMB1 insertion seemed to be driven largely by hydrophobic interactions, not only due to fatty acid tails but also the D-Phe sidechain. The converged, inserted PMB1 conformation (S4 Fig) structurally resembles that observed in NMR studies performed by *Mares et al* [26] which focused on the interaction between LPS and PMB1. In both cases, the majority of the ring structure remained bound to the phospholipid headgroups on the membrane surface, while the hydrophobic portion of the ring (residues D-Phe and Leu) adopted an embedded orientation.

In the case of the IM, we also observed a further possible role of the DAB residues in antimicrobial action, namely attraction of water towards the membrane core. Unlike the OM models, upon loss of the surface-bound state and PMB1-membrane insertion, the lipid lateral diffusion rate increased beyond the PMB1-free rate, correlating with peptide/water membrane penetration. Concomitant with this, we observed a remarkable thinning effect of >0.7 nm and increase in area per lipid in the IM as a result of PMB1 insertion, whilst the resultant cross-leaflet interdigitation of acyl tails is consistent with data from spin-labelling and X-ray diffraction reported by *Boggs et al* [29] and *Theretz et al* [30]. Since the PMB1 does not aggregate within the IM, but instead inserts as monomers, we may hypothesise on the basis of our collective observations that PMB1 disrupts the IM not through traditional mechanisms of pore formation but through membrane insertion, bilayer thinning, and water penetration. Such effects are certainly indicative of AMP permeation and the early stages of membrane disruption [34].

The limitations of the current study arise from the slow lateral diffusion of LPS, such that the long simulation times required to witness complete PMB1 translocation within the asymmetric LPS membrane is not presently feasible with atomistic simulations. Indeed it has recently been shown that even for simple, phospholipid bilayers, the simulation times required for convergence have historically been seriously underestimated [35]). The slow reorganization of ionic interactions involving zwitterionic phospholipid headgroups when solutes penetrate the lipid-water interface are a particular problem, which is accentuated in the complex LPS headgroups we are studying. Other studies have suggested that atomistic molecular dynamics simulations of AMPs require multi-microsecond timescales [36]. Our simulations reported here show that even non-equilibrium methods such as steered MD are not able to access insertion behaviour into membranes and thus another approach is needed. The large molecular systems and extended timescales accessible to coarse-grain molecular dynamics (CG-MD) simulations provide an alternative and complementary route to studying antimicrobial peptides. Indeed, CG-MD studies have been shown to provide insights into the action of antimicrobial peptides in flat lipid bilayers and spherical vesicles [15, 37, 38]. To study the OM of Gram-negative bacteria using this approach, a coarse-grain model of LPS is urgently needed. Nevertheless, the simulations we have performed have helped to identify the initial stages of PMB1 action on the OM of Gram-negative bacteria, and in particular highlight the regions of the peptide that form strongest interactions with the LPS molecules of the outer membrane. In future, a multiscale simulation approach based on these studies may provide further insights regarding mechanism of action.

In conclusion, in this study we have shed light on the potential mechanisms for bacterial envelope disruption by PMB1. The aggregation witnessed in the OM models is suggestive of the possible early stages of self-regulated translocation / pore formation, whilst the fatty acid tail insertion in the lipid A environment also appears to be dependent upon aggregation in order to create a charge-free area to allow PMB1 penetration, providing further evidence for a pore model of self-regulated uptake. We may speculate that a ‘ladder’ type mechanism occurs in the context of the full LPS membrane, with the PMB1 molecules initially aggregating on the surface, prior to further penetration through the sugar region by the DAB residues, subsequently disrupting the high surface charge density of the counterion-cross-linked lipid A moieties and resulting in fatty acid tail penetration within the hydrophobic membrane core. A stepwise process for PMB1 disruption of the IM has also been established, beginning with DAB-based adsorption and followed by rapid fatty acid tail insertion within the bilayer, supported by the hydrophobic D-Phe. In this case, deep penetration of monomeric PMB1 molecules enables the DAB residues to drag water into the membrane, suggesting an alternative antimicrobial mechanism for IM destabilisation. Nevertheless, it is possible that other mechanisms (e.g. carpet model) may apply at higher concentrations of PMB1.

## Methods

### Simulation Systems

All simulations systems are summarised in Table 1.

#### Lipid A bilayer

A symmetric lipid A bilayer, containing 16 lipid A molecules in each leaflet of the bilayer, was taken from our previous published study of the *E*. *coli* OM [27].

#### Outer membrane

The *E*. *coli* outer membrane model used in this work contained the minimal Re LPS (i.e. lipid A plus two 3-deoxy-D-manno-oct-2-ulosonic acid or KDO sugars) [39]. While *E*. *coli* mutants containing this level of LPS are far more susceptible to, for example, hydrophobic compounds [40], this level of LPS was deliberately chosen so as to maximize the likelihood of observing membrane disruption during the outer membrane simulations with polymyxin B1. The Re LPS asymmetric outer membrane was constructed as described previously for our models of *E*. *coli* Rd_1_ LPS (i.e. the Re LPS plus three further heptose sugars of the inner core) and *H*. *influenzae* I-69 Rd-/b+ LPS (lipid A plus one phosphorylated KDO) containing outer membranes [27, 41]. Briefly, two KDO molecules were added onto each lipid A molecule in the equilibrated symmetric lipid A membranes (described above). Simulations were subsequently performed for a further 500 ns to allow equilibration of these symmetric Re LPS membranes. An atomistic phospholipid inner leaflet of an appropriate size, containing 90% phosphatidylethanolamine (PE), 5% phosphatidylglycerol (PG) and 5% cardiolipin [42] (with the realistic 1-palmitoyl 2-cis-vaccenyl tails for PE and PG and 1-palmitoyl 2-cis-vaccenyl 3-palmitoyl 4-cis-vaccenyl for cardiolipin [43–45]), was constructed from a coarse-grained simulation and used to make an asymmetric outer membrane. This outer membrane was subjected to further simulation to allow the properties of the membrane to equilibrate. This structure was used as the starting structure for the Re LPS *E*. *coli* outer membrane simulations performed in this work.

#### Inner membrane

The IM was built by initially performing a coarse-grained simulation, using the Martini phospholipid force field [46, 47] for 1 microsecond to equilibrate a membrane containing 75% PE, 20% PG and 5% cardiolipin. To obtain the coordinates of the atomistic bilayer, the final structure from the coarse-grained simulation was reverse mapped to an atomistic resolution using the SUGAR-PIE methodology (extended to include these phospholipids) [48].

#### Polymyxin B1

The structure of polymyxin B1 [49, 50] was constructed using the Avogadro software [51]. To add polymyxin molecules to the equilibrated membrane structures, the systems were simply expanded in the *z* dimension (i.e. the axis perpendicular to the plane of the membranes) and polymyxin molecules were manually added to the systems using the Visual Molecular Dynamics (VMD) software [52]. In the simulations with twelve polymyxin molecules, the six original polymyxin molecules had already become bound to the membrane surface and an additional six polymyxin molecules were added at random positions above the membrane in a second step.

### Simulation Parameters and Protocol

All simulations performed in this work used the GROMACS molecular dynamics software [53, 54], version 4.5.1 [55]. Standard parameters taken from the GROMOS 53A6 force field [56] were used to model the polymyxin B1 molecule in its fully ionized state. The parameters for the LPS molecules were as described and used previously [27, 57] and the GROMOS-CKP (Chandrasekhar[58]-Kukol[59]-Piggot[27, 60]) parameters were used for the phospholipids. The SPC water model [61] was used in all simulations. During the simulations, the LPS, phospholipids and solvent (water plus counterions) were maintained at a constant temperature of 313 K using the Nosé-Hoover thermostat [62, 63] with a time constant of 0.5 ps. The only exception being the lipid A bilayer simulations which were performed at a temperature of 323 K. These temperatures were chosen as they are above the gel to liquid crystal phase transition temperatures of all the lipids used in the simulations [64–68]. A pressure of 1 bar was maintained using anisotropic pressure coupling with the Parrinello-Rhaman barostat [69, 70] and a time constant of 5 ps. Electrostatic interactions were treated using the smooth particle mesh Ewald (PME) algorithm [71] with a short-range cutoff of 0.9 nm. The van der Waals interactions were truncated at 1.4 nm with a long-range dispersion correction applied to the energy and pressure. The neighbor list was updated every five steps during the simulations. All bonds were constrained using the P-LINCS algorithm [72] allowing a 2 fs time step to be applied. All LPS-containing membrane systems were neutralized with Mg^2+^ ions, whereas the inner membrane model was neutralized with Na^+^ ions.

## Supporting Information

S1 Fig Center of mass of groups along the system z–axis, with subsequently added PMB1 in different colours as indicated (A—LPS, B—lipid A, C–IM). (TIF)

S2 Fig Solvent accessible surface area of PMB1 throughout the simulation. Comparing the charged ring portion (green) to the hydrophobic tail portion (red), with subsequently added PMB1 in different colours as indicated (A—LPS, B—lipid A, C—IM).
(TIF)

S3 Fig Center of mass of the DAB residues relative to the rest of the peptide during the binding portion of the simulation. (TIF)

S4 Fig Radial distribution functions along the z–axis showing solvent penetration relative to the membrane center of mass (top row A—C). Cumulative distribution of solvents in the z–axis relative to the membrane center of mass (middle row D—F). Radial distribution function (all axis) showing solvent proximity to the DAB amine (bottom row F—I)
(TIF)

S5 Fig A low concentration simulation of two PMB1 molecules in LPS that show aggregation within 100 ns, as seen in simulations with higher PMB1 concentration (PMB1—blue, LPS head groups—grey/red, LPS tails—orange. (TIF)

S6 Fig Snapshots of the system after 200 ns (left) and 600 ns (right) when divalent cations are replaced by monovalent cations prior to simulation. The peptides are cyan, the LPS-containing outer leaflet is yellow and the phospholipids of the inner leaflet are orange.
(TIF)

S7 Fig Mass density profiles for Lipid A and LPS (A—LPS, B—lipid A). (TIF)

S8 Fig Bilayer thickness plots, blue areas corresponding to thin areas and red and yellow corresponding to thicker areas (Starting thickness plot—left, final thickness plot—right). (TIF)

S9 Fig Deuterium order parameters of the inner membrane with the PMB1–free order parameters in red and the order parameters after this system has been exposed to PMB1 for 3345 ns in black. (TIF)

S10 Fig The position of Polymyxin B1 (yellow) relative to the phospholipids (head group by colour, tails in pale blue) in our simulations (top) (TIF)

S1 Table The role of DAB in membrane binding. (DOCX)

S2 Table Peptide-lipid head group hydrogen bonding. (DOCX)

S3 Table Lateral diffusion of the membrane lipids. (DOCX)

S4 Table Average membrane thickness of membranes, measured between the center of mass of the headgroups in all cases. (DOCX)
