# Supplementary figures and images for: Interaction of the Antimicrobial Peptide Polymyxin B1 with Both Membranes of E. coli: A Molecular Dynamics Study
Source: PLoS Comput Biol. 2015 Apr 17;11(4):e1004180. doi: 10.1371/journal.pcbi.1004180 (PMC4401565; doi:10.1371/journal.pcbi.1004180)

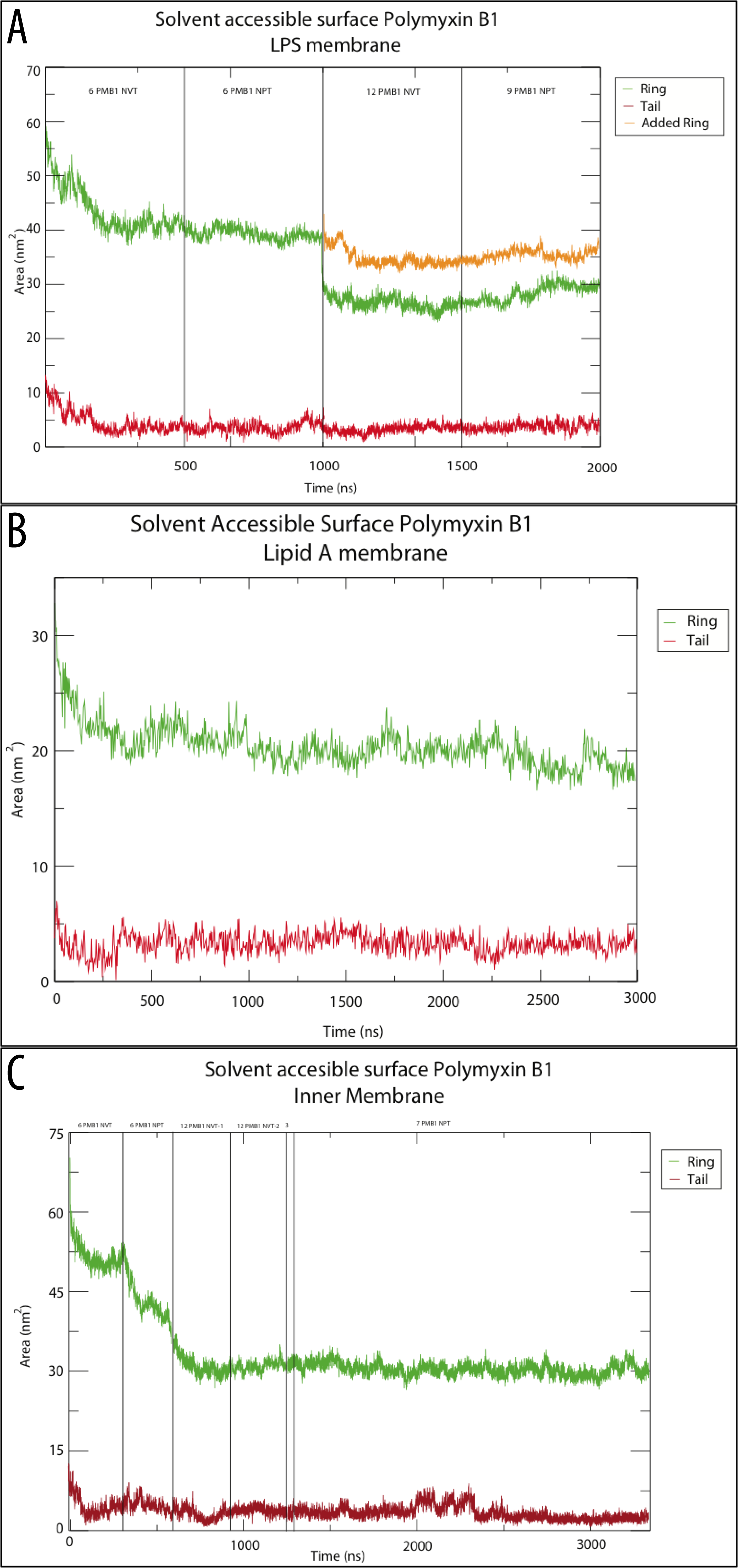

Supplement: S2 Fig — Comparing the charged ring portion (green) to the hydrophobic tail portion (red), with subsequently added PMB1 in different colours as indicated (A—LPS, B—lipid A, C—IM). (TIF) [file pcbi.1004180.s002.tif]

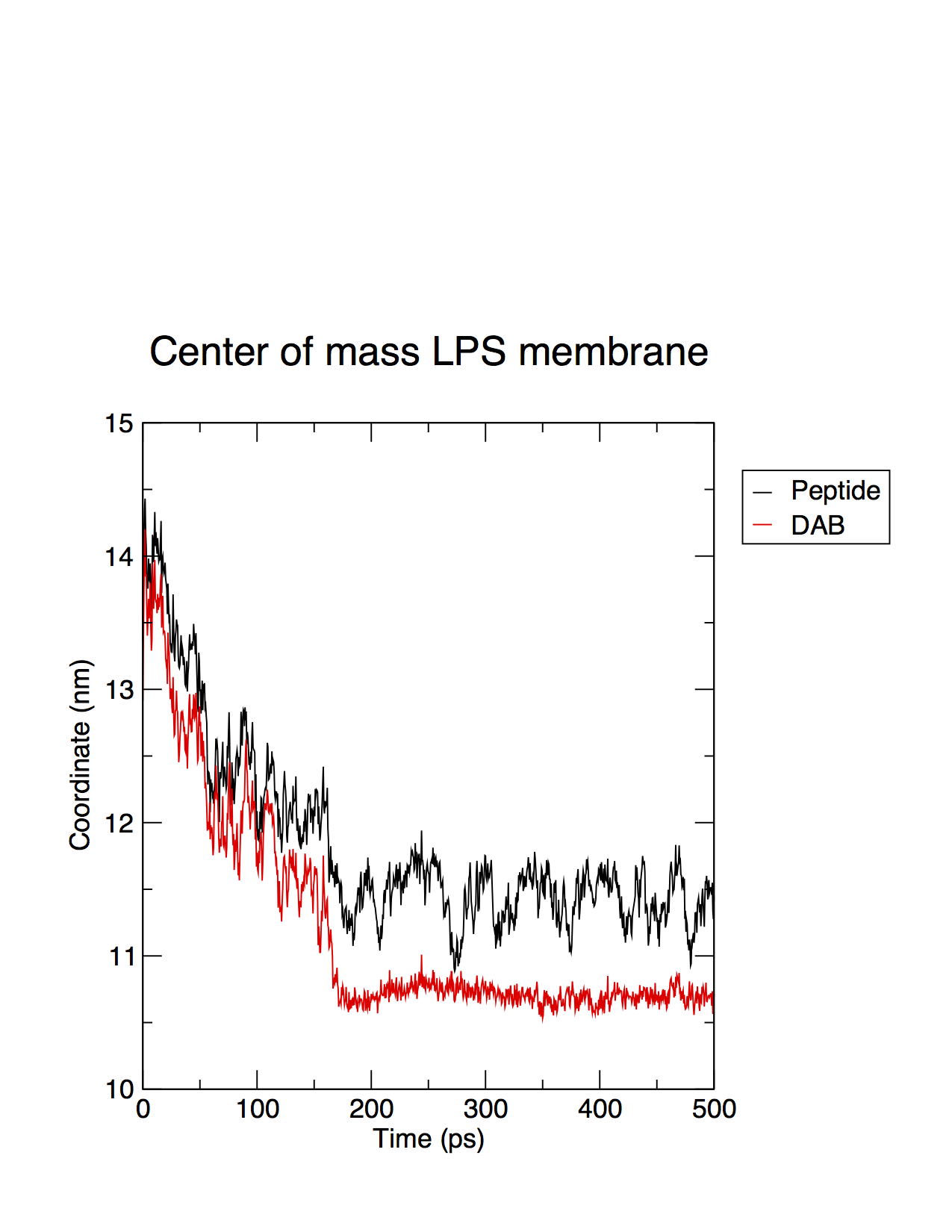

Supplement: S3 Fig — (TIF) [file pcbi.1004180.s003.tif]

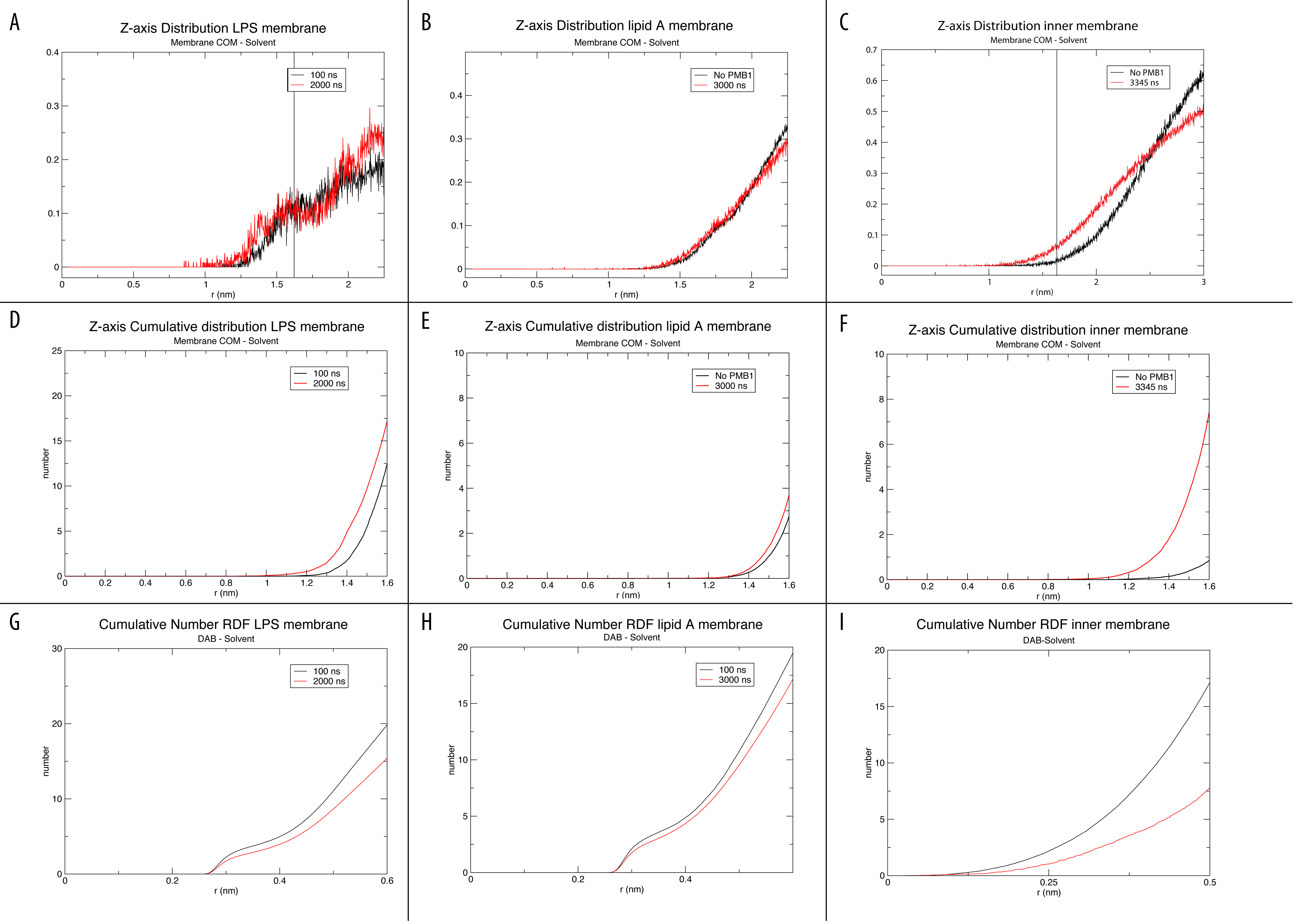

Supplement: S4 Fig — Cumulative distribution of solvents in the z–axis relative to the membrane center of mass (middle row D—F). Radial distribution function (all axis) showing solvent proximity to the DAB amine (bottom row F—I) (TIF) [file pcbi.1004180.s004.tif]

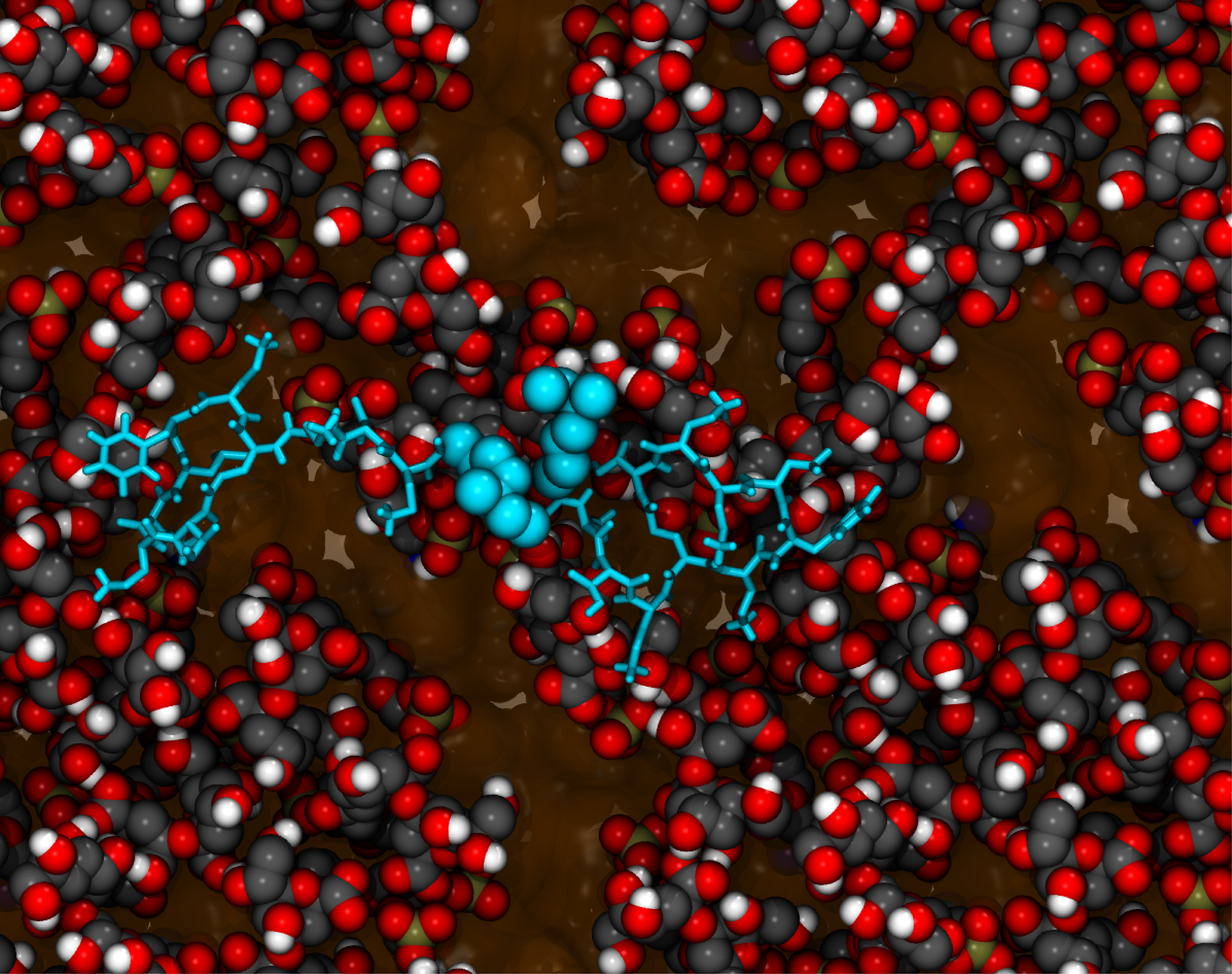

Supplement: S5 Fig — (TIF) [file pcbi.1004180.s005.tif]

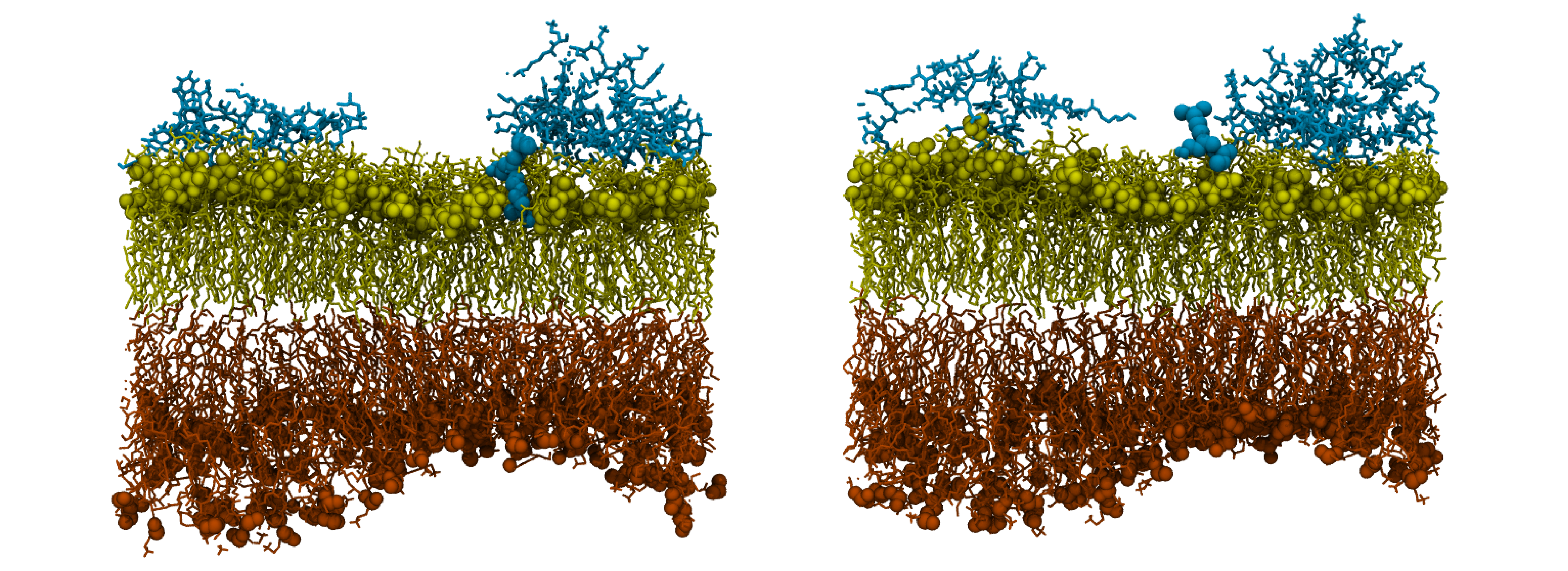

Supplement: S6 Fig — The peptides are cyan, the LPS-containing outer leaflet is yellow and the phospholipids of the inner leaflet are orange. (TIF) [file pcbi.1004180.s006.tif]

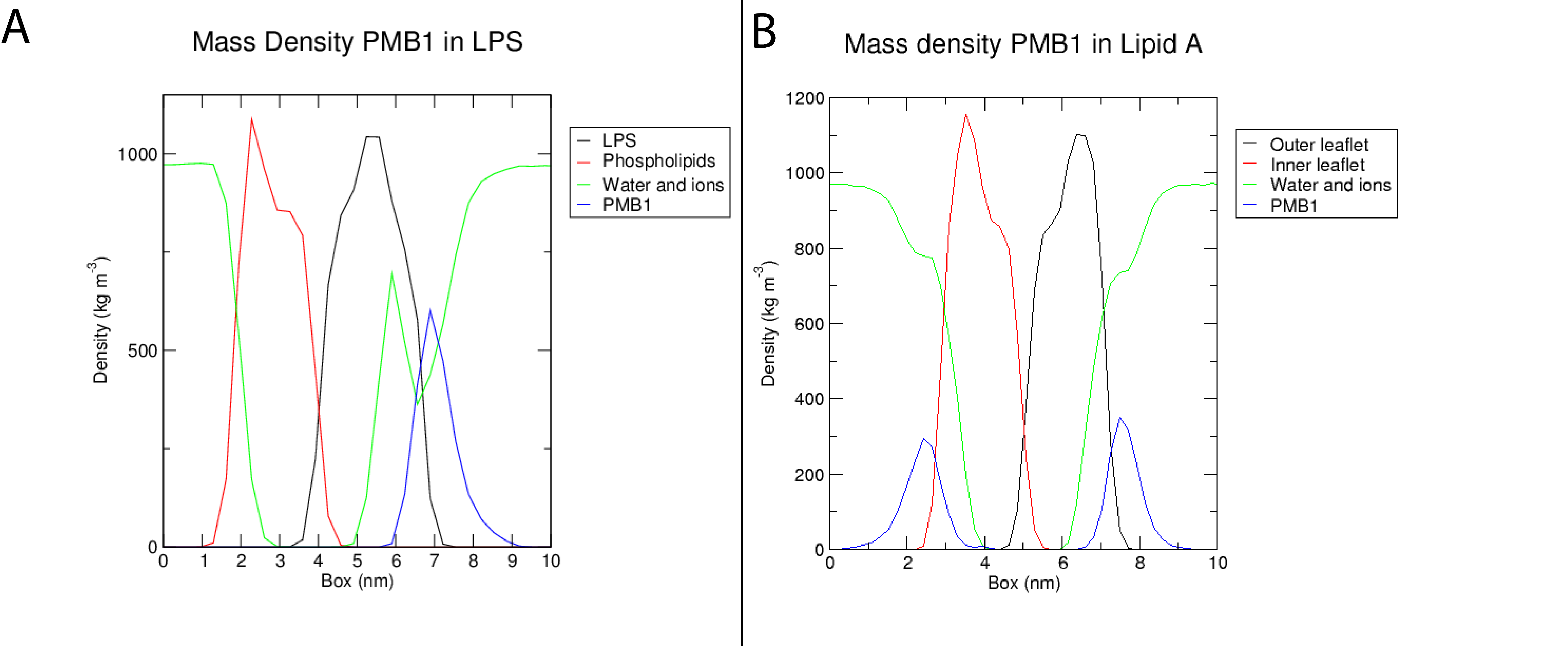

Supplement: S7 Fig — (TIF) [file pcbi.1004180.s007.tif]

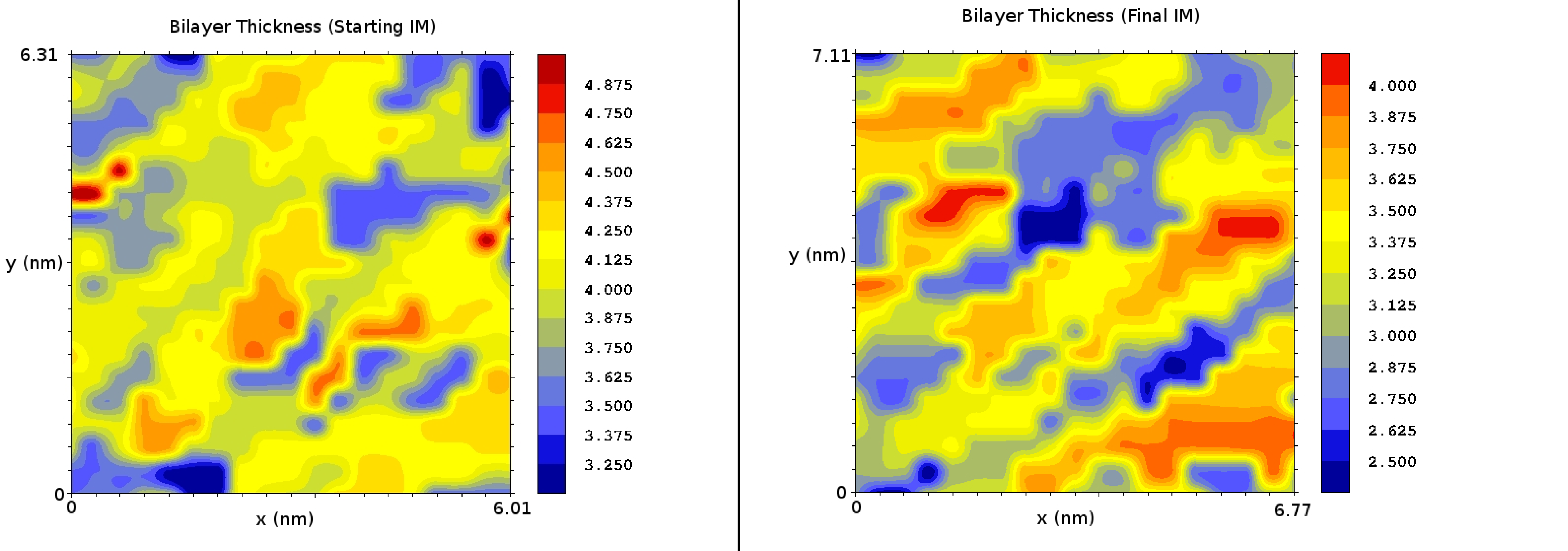

Supplement: S8 Fig — (TIF) [file pcbi.1004180.s008.tif]

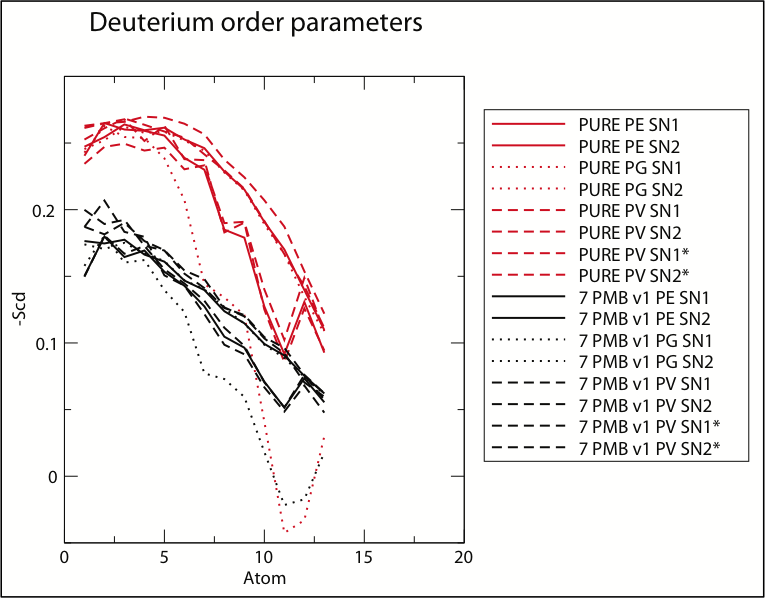

Supplement: S9 Fig — (TIF) [file pcbi.1004180.s009.tif]

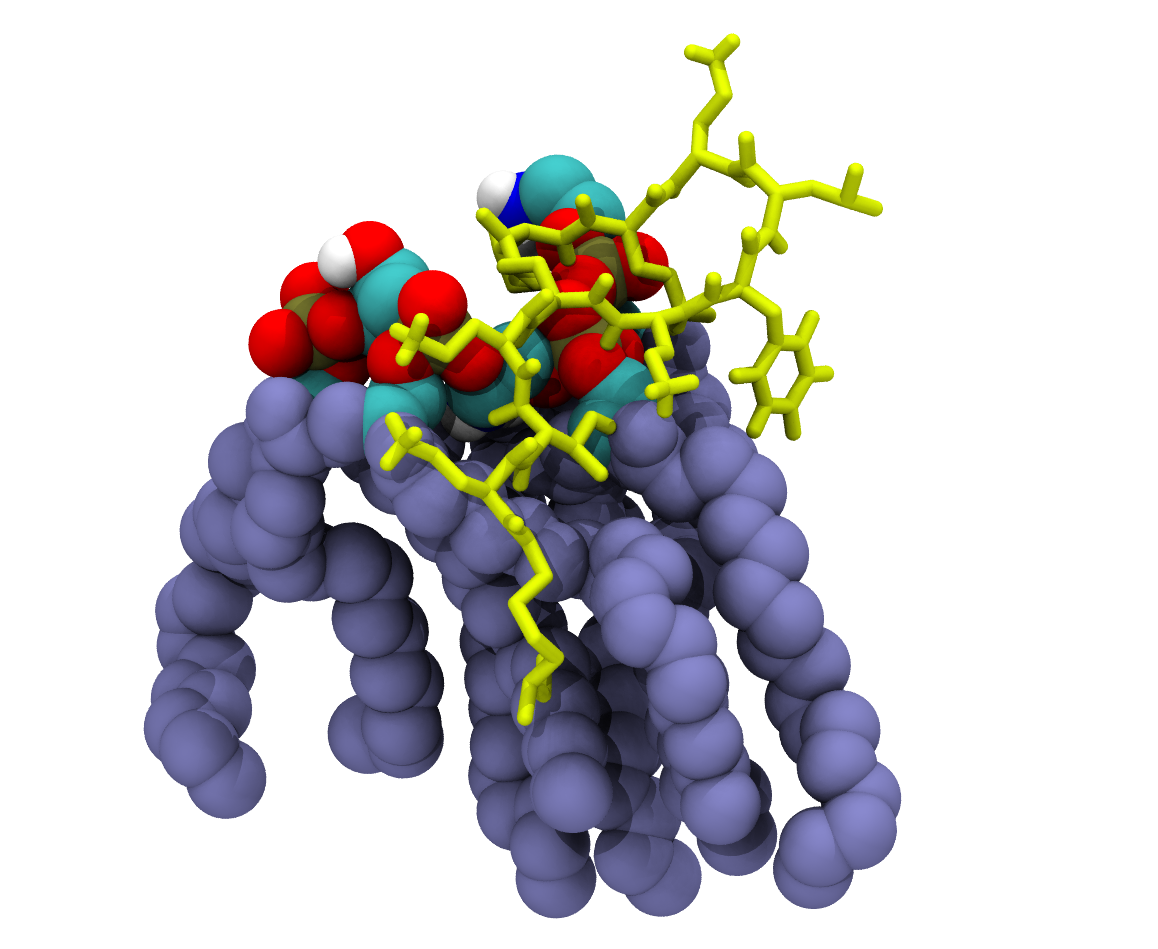

Supplement: S10 Fig — (TIF) [file pcbi.1004180.s010.tif]
