# Supplementary material for: Interaction of the Antimicrobial Peptide Polymyxin B1 with Both Membranes of E. coli: A Molecular Dynamics Study
Source: PLoS Comput Biol. 2015 Apr 17;11(4):e1004180. doi: 10.1371/journal.pcbi.1004180 (PMC4401565; doi:10.1371/journal.pcbi.1004180)
Supplement: S1 Table — (DOCX) [file pcbi.1004180.s011.docx]

| Simulation | Initial Peptide-Lipid Interaction/ DAB-Lipid Interaction | Percentage of total interaction | Final Peptide-Lipid Interaction/ DAB-Lipid Interaction | Percentage of total interaction |
| --- | --- | --- | --- | --- |
| LPS | 39.28/16.72 | 42.57% | 72.97/39.66 | 50.23% |
| Lipid A | 30.52/21.99 | 72.03% | 52.23/33.01 | 63.20% |
| IM | 40.25/18.62 | 46.27% | 53.12/9.05 | 17.06% |

**Table 1 - The role of DAB in membrane binding.**
