# Supplementary material for: Interaction of the Antimicrobial Peptide Polymyxin B1 with Both Membranes of E. coli: A Molecular Dynamics Study
Source: PLoS Comput Biol. 2015 Apr 17;11(4):e1004180. doi: 10.1371/journal.pcbi.1004180 (PMC4401565; doi:10.1371/journal.pcbi.1004180)
Supplement: S2 Table — (DOCX) [file pcbi.1004180.s012.docx]

| Simulation | Number of peptide-lipid hydrogen bonds at any one time | Hydrogen bonds per PMB1 Monomer |
| --- | --- | --- |
| LPS | 72.97 (+/- 7.32) | 8.11 |
| Lipid A | 52.24 (+/- 2.16) | 6.53 |
| IM | 69.89 (+/- 3.01) | 9.93 |

**Table 2 - Peptide-lipid head group hydrogen bonding.**
