# Supplementary material for: Interaction of the Antimicrobial Peptide Polymyxin B1 with Both Membranes of E. coli: A Molecular Dynamics Study
Source: PLoS Comput Biol. 2015 Apr 17;11(4):e1004180. doi: 10.1371/journal.pcbi.1004180 (PMC4401565; doi:10.1371/journal.pcbi.1004180)
Supplement: S3 Table — (DOCX) [file pcbi.1004180.s013.docx]

| System | Starting Lateral Diffusion (10^-8^ cm^2^/s) | Final Lateral Diffusion (10^-8^ cm^2^/s) |
| --- | --- | --- |
| IM | 6.63 (± 0.72) | 5.01 (± 0.02) |
| Lipid A | 0.29 (± 0.042) | 0.065 (± 0.031) |
| LPS | 0.12 (± 0.0071) | 0.082 (± 0.011) |

**Table 4 – Lateral diffusion of the membrane lipids.**
