# Supplementary material for: Interaction of the Antimicrobial Peptide Polymyxin B1 with Both Membranes of E. coli: A Molecular Dynamics Study
Source: PLoS Comput Biol. 2015 Apr 17;11(4):e1004180. doi: 10.1371/journal.pcbi.1004180 (PMC4401565; doi:10.1371/journal.pcbi.1004180)
Supplement: S4 Table — (DOCX) [file pcbi.1004180.s014.docx]

| System | Starting Average Thickness (nm) | Final Average Thickness (nm) |
| --- | --- | --- |
| IM | 3.99 (± 0.034) | 3.28 (± 0.12) |
| Lipid A | 3.74 (± 0.024) | 3.74 (± 0.050) |
| LPS | 4.03 (± 0.013) | 3.93 (± 0.11) |

**Table 5 - Average membrane thickness of membranes, measured between the center of mass of the headgroups in all cases.**
